# Haptoglobin and Pig-MAP Levels in Serum, Milk and Saliva in Healthy Sows Before and After Parturition

**DOI:** 10.3390/life16050783

**Published:** 2026-05-07

**Authors:** Marina Sigg, Matilde Piñeiro, Sonja Hartnack, Barbara Riond, Dolf Kümmerlen, Wolfgang Pendl

**Affiliations:** 1 Division of Swine Medicine, Department of Farm Animals, Vetsuisse Faculty, University of Zurich, 8057 Zurich, Switzerland; dkuemmerlen@vetclinics.uzh.ch; 2 Acuvet Biotech, C/Bari 25, 50197 Zaragoza, Spain; 3 Clinical Department for Livestock and Transformation of Food Systems, Centre for Veterinary Public Health and One Health, University of Veterinary Medicine Vienna, 1210 Vienna, Austria; 4 Clinical Laboratory, Department for Clinical Diagnosis and Services, Vetsuisse Faculty, University of Zurich, 8057 Zurich, Switzerland; 5 Veterinary Practice Pendl, 8083 St. Stefan im Rosental, Austria

**Keywords:** haptoglobin, pig-MAP, acute phase proteins, sows, parturition, rectal temperature

## Abstract

Background: Various acute phase proteins (APPs) are already used as diagnostic biomarkers or are considered promising candidates. The aim of this study was to measure the concentrations of haptoglobin and pig major acute phase protein (pig-MAP) in the serum, milk and saliva of healthy sows before and after parturition. Methods: The measurements from 19 sows were included in the evaluation. Samples were taken at seven time points including T0 (112th day of pregnancy), T1 (intrapartum/birth of the first piglet), T2 (12 h postpartum), T3 (24 h postpartum), T4 (48 h postpartum), T5 (72 h postpartum) and T6 (14 d postpartum), and general condition parameters, such as the rectal temperature, were recorded. The samples were analysed using ELISA kits. The correlation between the APPs in different body fluids was investigated. Results: For serum and saliva concentrations, time points T1–T6 were compared to time point T0 to detect changes in APP concentration compared to prepartum levels. The pig-MAP levels in serum at time points T3–T5 were significantly increased compared to T0; the haptoglobin levels at time points T2–T5 were significantly increased compared to T0; and the rectal temperature was significantly increased at all later time points compared to T0. Comparison of T0–T5 to T6 (milk T1–T5 to T6) revealed that pig-MAP in serum, at time points T4 and T5, and haptoglobin levels, at T3–T5, were significantly different compared to T6. For both APPs in milk, the concentrations at all time points (T1–T5) were significantly elevated compared to T6. In saliva, there was a significant increase at T5 compared to T6 for haptoglobin. Conclusions: This descriptive pilot study provides insights into the magnitude, timing and significance of the increase in APP levels in sows during and after parturition and can be used as a basis for future studies on the early detection and diagnosis of diseases during this period.

## 1. Introduction

Acute phase proteins (APPs) are produced by the body during an acute phase reaction. When tissue is injured or bacterial toxins are present, cytokines are released locally, primarily by monocytes. Different cytokines are known to act both locally and systemically; they reach the liver via the bloodstream and induce the synthesis of APPs by hepatocytes. APPs can be categorised as either positive or negative. The concentrations of positive APPs (e.g., haptoglobin and pig major acute phase protein (pig-MAP)) rise sharply during the acute phase reaction, while the concentrations of negative APPs fall. The acute phase reaction can be measured for several days but is highly dependent on the species and the extent of tissue damage. In chronic inflammation, APPs are permanently elevated, but usually somewhat lower than with acute stimuli [1].

Different APPs are already used as biomarkers in diagnostics or are considered as potential candidates. Due to their high response in animals and short half-life in serum, APPs can be used as indicators to assess the health status of livestock for diagnostic, prognostic or monitoring purposes [1].

The most common APPs used for dogs, cats, pigs, horses and ruminants are serum amyloid A (SAA), C-reactive protein (CRP), haptoglobin (Hp), major acute phase protein (MAP) and α-1-acid glycoprotein (AGP) [2].

Various studies have shown that haptoglobin and pig-MAP could be suitable indicators of health status in pigs [1]; for example, biomarkers of health status, especially around birth, would be helpful in recognising diseases such as postpartum dysgalactia syndrome (PPDS) at an early stage. However, reference values need to be defined first.

Multiple factors that influence the concentration of APPs are known and have been described in the literature, which can be divided into physiological and pathological factors: physiological factors influencing serum pig-MAP and haptoglobin levels include breed, sex [3,4], age [5,6], feeding regime [7], pregnancy, birth and lactation [8,9,10].

It is known that various disorders increase serum pig-MAP and haptoglobin concentrations. Examples include postweaning multisystemic wasting syndrome (PMWS) [11], porcine reproductive and respiratory syndrome virus (PRRSV) infection [12], Aujeszky’s disease [13], African swine fever [13] and *Streptococcus suis* serotype 2 infection [14] for pig-MAP, and *Porcine Circovirus 2* (PCV-2) Systemic Disease [15], PRRSV [12] and *Streptococcus suis* serotype 2 infections [14], Aujeszky’s disease and African swine fever virus infection [13] for haptoglobin. PPDS [15], lameness, ear and tail biting, diarrhoea and respiratory diseases can also lead to increases in serum concentrations of haptoglobin [16].

Other factors that increase the serum concentrations of haptoglobin and pig-MAP include surgery, experimentally induced sterile inflammation and experimental infections with *Actinobacillus pleuropneumoniae* (serotypes 1, 2 and 5), *Mycoplasma hyorhinis* and *Toxoplasma gondii* [1].

There are few studies on haptoglobin levels in milk. One study described an increase in haptoglobin levels in milk around parturition [17].

Several studies have shown that diseases in pigs, such as PRRSV infections [18,19], respiratory diseases, diarrhoea, lameness, and multiple abscesses and/or hernias [20], can lead to an increase in haptoglobin in saliva.

APPs may have potential as indicators for sow health around farrowing. For example, while the potential risk of PPDS can be evaluated by measuring rectal temperature, APPs could provide a more precise evaluation and risk assessment for PPDS. In this context, PPDS is also associated with systemic inflammatory processes that share common pathophysiological mechanisms with Swine Inflammation and Necrosis Syndrome (SINS), a condition characterized by inflammatory lesions affecting the tail, ears, claws, and teats in piglets [21,22]. These similarities suggest that acute phase proteins (APPs) may also be affected in SINS and could potentially serve as indicators for early detection [23].

The aim of this pilot study was to describe the course of haptoglobin and pig-MAP changes in the milk, serum and saliva of healthy sows before and after parturition.

## 2. Materials and Methods

### 2.1. Animals

Since no comparable studies were available, in this trial, the sample size was set at 24. The landrace hybrid sows were aged between 11 months and 3.5 years and were in their first to seventh lactation with a body condition score (BCS) of 3–4. They were clinically examined to exclude PPDS and sampled pre-, peri- and postpartum. The sows were housed at the pig facility of the Vetsuisse Faculty of the University of Zurich. The animals received a commercial complete feed (UFA 463-7 Universal IPS, UFA AG, Herzogenbuchsee, Switzerland). The feed contained (per kg) 155 g crude protein, 40 g crude fat, 80 g crude fibre, and 60 g crude ash. The main ingredients included barley, wheat bran, sugar beet pulp, soybean meal, linseed, molasses, oat hulls, mineral supplements, and animal fat.

The diet was supplemented with vitamins and trace elements, including vitamin A (10,350 IU/kg), vitamin D_3_ (850 IU/kg), and vitamin E (21 mg/kg), as well as copper (15 mg/kg), zinc (100 mg/kg), manganese (50 mg/kg), iodine (1 mg/kg), and selenium (0.4 mg/kg). Propionic acid (1000 mg/kg) was included as a preservative. Animals had ad libitum access to fresh water.

The birth of all sows was induced by 0.175 mg of cloprostenol and occurred within a time frame of between 120 and 510 min. One animal showed clear clinical signs of PPDS during the study period and was therefore excluded from the analysis.

Four further sows were excluded due to lack of blood, milk and saliva samples. A total of 19 sows were therefore used for the statistical analysis.

The age of the sow, the number of litters, the duration of birth and the BCS at T0 or T1 were recorded, which are summarised in Appendix A (Table A1).

### 2.2. Clinical Examination and Samples

Samples were taken and clinical examinations were carried out at seven different time points: T0 (112th day of pregnancy), T1 (intrapartum/birth of the first piglet), T2 (12 h postpartum), T3 (24 h postpartum), T4 (48 h postpartum), T5 (72 h postpartum) and T6 (14 d postpartum).

Clinical examinations included general behaviour, breathing rate, rectal temperature, appetite, udder colour, udder consistency and faecal consistency using the scale of Oliviero et al. (Table 1) [24]. The temperature of the udder cranially, medially and caudally was recorded using a Fluke^®^ 64 max infrared thermometer (Fluke Europe B.V., 5602 BD Eindhoven, The Netherlands). No lasting changes in the clinical condition of the animals that would indicate illness were observed. More than two of the main parameters—general condition, appetite, udder consistency, stool consistency and temperature—were only altered at one time point for one sow. No severe alterations occurred. Thus, only short-term clinical changes in individual vital parameters were observed.

At T0–T6, blood was collected from the jugular vein into a 9 mL Sarstedt S-Monovette^®^ Serum tube (Ehrhardt Medizinprodukte GmbH, 73312 Geisslingen/Steige, Germany) and then centrifuged at 2000× *g* for 10 min to obtain serum. The serum was heat-inactivated at 56 °C for 30 min and stored at −20 °C until further analysis. A total of 4–8 mL of milk was collected by hand and for the saliva samples, the animals were placed in a snare and cotton swabs were inserted using a clamp. The concentrations of haptoglobin and pig-MAP were then determined in both substrates. No milk samples could be collected at T0 since the sows were not lactating at this time. Samples were stored at −20 °C until analysis. The blood and milk samples were analysed using commercially available test kits (Haptoglobin Kit PHASE RANGE^®^, Tridelta Development Ltd., catalogue No. TP801 (Maynooth, County Kildare, Ireland); PigMAP Kit ELISA^®^, Acuvet biotech, AC/PME (Zaragoza, Aragón, Spain)). Saliva samples were analysed by ELISA using a commercially available ELISA kit (Acuvet ELISA pig Haptoglobin, Acuvet Biotech (Zaragoza, Aragón, Spain)). Although no new ELISA validation was performed in this study, the assays used have been previously described in studies such as Tecles et al. (2007) and Nielsen et al. (2004) [25,26]. Only haptoglobin was measured in the saliva samples, as pig-MAP is not stable in saliva during storage.

### 2.3. Statistics

The data were statistically analysed using R 4.5.1 (R Core Team, 2025) [27] in combination with RStudio (RStudio Team, 2025.05.1+513) [28]. In addition to descriptive statistics, Pearson’s correlation tests were performed to compare serum, milk and saliva concentrations. These analyses were performed for each time point and for the entire study period.

A linear mixed model with the animal as a random effect and Dunnett’s model approach was used as a correction method to compare T1–T6 with T0. Time (T0–T6) was treated as a categorical fixed effect. The individual animal (ID) was included as a random intercept to account for repeated measurements within subjects. The model can be expressed as Y_{ij} = β0 + β1 Day_{ij} + u_i + ε_{ij} (Y_{ij} = measurement for animal i at day j, β0 = intercept, β1 = effect of day, u_i = random intercept for animal i, ε_{ij} = residual). Model assumptions were assessed using residual diagnostics and showed no relevant deviations from normality or homoscedasticity. Since no values could be determined for milk at T0, the same procedure as the one for the comparison of serum concentrations was used, and T6 was used as the basis for comparison for T1–T5. Using this approach, comparisons could also be made for the APP concentrations in milk.

All means ± standard deviations and medians are shown in Appendix A in Table A2. Statistical significance was set at *p* < 0.05, with ** indicating *p* < 0.01 and *** indicating *p* < 0.001.

## 3. Results

### 3.1. Serum and Saliva Concentrations of Pig-MAP and Haptoglobin at T1–T6 Compared with T0

Serum pig-MAP concentrations were significantly higher than T0 at T3 (*p* < 0.004), T4 (*p* < 0.001) and T5 (*p* < 0.001). Although the difference between T2 and T0 was not significantly different, an increasing trend could be observed (*p* < 0.087).

For haptoglobin, significant differences were seen between the serum concentrations at T2 (*p* < 0.002), T3 (*p* < 0.001), T4 (*p* < 0.001) and T5 (*p* < 0.001) compared to T0. Thus, a significant increase was detected even earlier in haptoglobin serum concentrations compared to pig-MAP concentrations.

**Figure 1:**
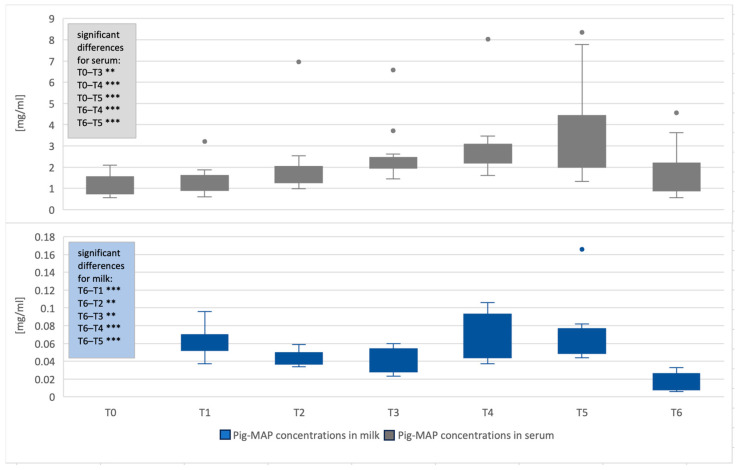
Concentrations of pig-MAP in serum and milk over the entire period [mg/mL]. The boxes represent the first to third quartiles, with the line marking the median. The minimum and maximum values outside the first and third quartiles are marked as whiskers (limited to a maximum of 1.5 times). Significance codes in boxes: <0.001 ‘***’ and <0.01 ‘**’ compared to T0 or T6; timepoints: T0 (112th day of gestation), T1 (intrapartum), T2 (12 h postpartum (p.p.)), T3 (24 h p.p.), T4 (48 h p.p.), T5 (72 h p.p.) and T6 (14 d p.p.).

**Figure 2:**
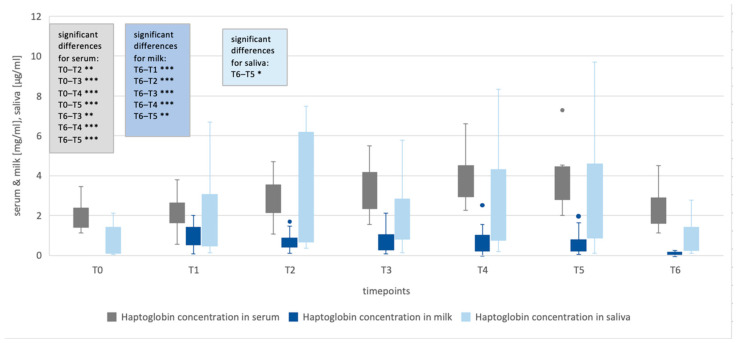
Concentrations of haptoglobin in serum, milk [mg/mL] and saliva [µg/mL] over the entire study period. The boxes represent the first to third quartiles, with the line marking the median. The minimum and maximum values outside the first and third quartiles are marked as whiskers (limited to a maximum of 1.5 times). Outlier saliva levels are not shown. Significance codes in boxes: <0.001 ‘***’, <0.01 ‘**’ and <0.05 ‘*’ compared to T0 or T6; timepoints: T0 (112th day of gestation), T1 (intrapartum), T2 (12 h postpartum (p.p.)), T3 (24 h p.p.), T4 (48 h p.p.), T5 (72 h p.p.) and T6 (14 d p.p.).

No significant differences were observed in haptoglobin levels in saliva at the different time points (Figure 1 and Figure 2, and Table A3 in Appendix A).

### 3.2. Rectal Temperature at T1–T6 Compared with T0

The rectal temperature was significantly higher at all time points after birth compared to before birth (*p* < 0.001) except at T1 (Table A3 in Appendix A). Over the entire study, 12 rectal temperature values above 39.5 were measured.

### 3.3. Pig-MAP and Haptoglobin in Serum, Saliva and Milk in T1–T5 Compared to T6

Milk concentrations at T1–T5 were compared to those at T6 since the animals were not lactating at T0. Compared to T6, all time points showed significantly higher levels of both APPs (*p* ≤ 0.006).

The concentrations of pig-MAP in serum were significantly higher at T4 (*p* < 0.001) and T5 (*p* < 0.001) compared to T6. Serum haptoglobin already differed significantly at T3 (*p* = 0.002) and, like pig-MAP, at T4 (*p* < 0.001) and T5 (*p* < 0.001) compared with T6.

Haptoglobin concentrations in saliva were significantly higher at T5 than at T6 (*p* = 0.018) (Figure 1 and Figure 2, and Table A4 in Appendix A).

### 3.4. Correlation Between Serum, Saliva and Milk Concentrations

The correlations were determined for the serum, milk and saliva concentrations of the two APPs. For pig-MAP, there was a significant correlation between milk and serum concentrations at T5 (*p* < 0.001 ***), T6 (*p* = 0.021 *) and for all time points (*p* < 0.001 ***). There was a significant correlation between serum and milk haptoglobin concentrations at T4 (*p* = 0.025 *) and for all time points (*p* = 0.031 *). All the results are shown in Appendix A in Table A5.

### 3.5. Natural Variation in Concentrations of the APPs over Time

**Figure 3:**
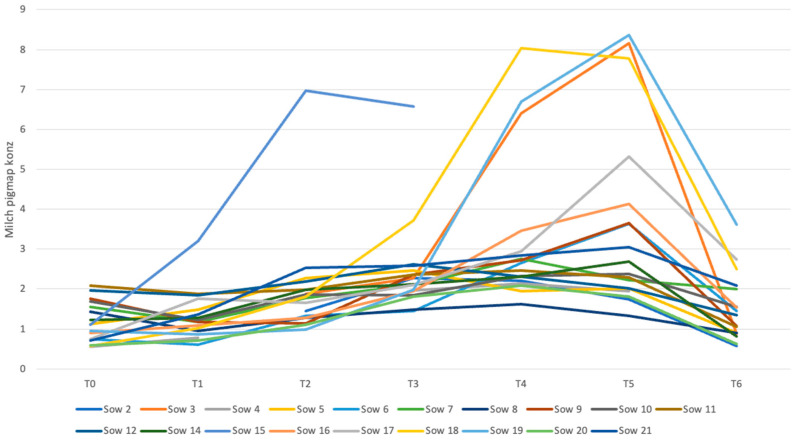
Trends in serum concentration of pig-MAP of each sow over the entire period [mg/mL].

A comparison of the trajectories of the APP concentrations in each individual sow revealed similarities. Although all sows were clinically classified as healthy, some animals showed higher concentrations compared to most of the other sows. Sows 3, 18 and 19 are particularly noteworthy as they had elevated rectal temperatures above 39.5 °C on two and three occasions, respectively. Sow 15 never recorded a rectal temperature above 39.5 °C. Figure 3 shows the changes in serum pig-MAP concentrations over the study period.

## 4. Discussion

### 4.1. Serum APP Concentrations

The measured serum concentrations at time T0 in this study correspond to the blood values of an adult sow prior to birth. When comparing the pig-MAP and haptoglobin serum concentrations at T0 with the results described in the literature for healthy, non-pregnant adult pigs by Clapperton et al. (2007), Piñeiro et al. (2009) and Saco et al. (2016), the values measured in this study were above their reference ranges [3,4,12]. Studies examining pregnant sows before and after birth, such as Piñeiro et al. (2006), also found pig-MAP concentrations above 1 mg/mL in sows on day 110 of gestation [8]. Our results are also comparable to those of Nguyen et al. (2022), who reported higher concentrations of pig-MAP and haptoglobin before birth than at the end of lactation [9]. They are also comparable to the data of Wierzchosławski et al. (2018), who reported an increase in acute phase proteins before birth in animals that developed postpartum dysgalactia syndrome [10].

Kaiser et al. (2018) [15] also investigated haptoglobin serum concentrations in sows before and after parturition. The serum concentrations at −60 to −36 h p.p. were within the standard deviation of the T0 values in this study. The values at time points 0 to 12 h p.p., 12 to 24 h p.p. and 24 to 36 h p.p. were also within the standard deviation of the concentrations at time points T1, T2 and T3. These similarities between the values in the study by Kaiser et al. (2018) [15] and in our own study also suggest that the 112th day of gestation cannot be compared with a healthy, non-pregnant sow, since APP levels are already elevated at this stage. Possible causes for this could be changes due to the preparation phase, suckling and milk production, or hormonal changes.

These findings suggest that inflammatory processes may already be occurring in the sow by the 112th day of pregnancy (T0), and that the serum concentrations of pig-MAP and haptoglobin found in our study cannot be compared with those of healthy, non-pregnant pigs [3,4,11,12].

Furthermore, genetics, age and sex may have influenced haptoglobin and/or pig-MAP levels in these studies, making direct comparisons difficult. The exact cause of this increase in APP levels prior to birth was not investigated in this study and could be the aim of future studies. Future studies using healthy as well as diseased sows should investigate to what extent an increase in APP concentration can still be considered physiological.

### 4.2. Milk APP Concentrations

The study of Hiss-Pesch et al. (2011) [17] can be used as a point of reference for haptoglobin concentrations in milk before and after parturition. In this study, the colostrum and milk concentrations at 12 h p.p. were within the reference values for T1 in the present study.

At the time of weaning (30 d p.p.), the values from the study by Hiss-Pesch et al. (2011) were higher than the T6 (14 d p.p.) values in the present study [17]. Therefore, the similar haptoglobin concentrations in milk in this study and in the study by Hiss-Pesch et al. (2011) indicates that the reference values for haptoglobin in milk should be in the range of 1.008 ± 0.58 mg/mL at T1 and 0.69 ± 0.42 mg/mL at T2 [17].

No investigations of pig-MAP concentrations in milk were found in the literature. The clearly significant increase in pig-MAP as well as haptoglobin levels at birth and up to 72 h p.p. compared to time point T6 (14 days p.p.) shows that these two APPs increase clearly and rapidly in milk and may therefore serve as indicators for postpartum diseases such as PPDS. The exact timing of the drop in milk concentrations still needs to be determined more precisely and could serve as an indicator to differentiate healthy from subclinical diseased sows.

### 4.3. Saliva Haptoglobin Concentrations

As haptoglobin levels in saliva have not previously been measured in peripartum sows, there are no results in the literature available for comparison. A.M. Gutiérrez et al. (2009) [18] examined healthy and sick pigs at different ages. The haptoglobin levels in healthy animals (0.8–2.04 mg/mL) are most comparable to the values at T6 (14 d p.p.; 1.68 ± 1.09 mg/mL) in this study. Clinically diseased animals (PRRSV *Mycoplasma hyopneumoniae*) had elevated values comparable to those at T1 (intrapartum). Accordingly, the values in our study in sows after birth were higher than in the study by A.M. Gutiérrez et al. (2009) [18]. This also indicates the occurrence of inflammatory events prior to birth. In L. Soler et al. (2013), healthy sows showed values comparable with those at T6 (14 d p.p.). Diseased animals showed significantly increased levels of haptoglobin in saliva, comparable to the sows in our study at time points T2–T5 [19]. In the study by Jaime Gómez-Laguna et al. (2010), animals that were experimentally infected with PRRSV did not show as high of an increase in haptoglobin in saliva as the sows in our study after giving birth [20].

Further studies are needed on haptoglobin in saliva, especially with a diseased control group, to determine how high concentrations can rise after giving birth.

### 4.4. Serum and Saliva APP Concentrations and Rectal Temperature at T1–T6 Compared with T0

The significant increase in serum concentrations of pig-MAP at the time points T3–T5 indicates a clear inflammatory reaction after birth. Haptoglobin serum concentrations also increased significantly compared to T0. However, the haptoglobin serum concentrations were already significantly increased at T2, while pig-MAP concentrations only showed a trend. There was no significant increase in haptoglobin in saliva compared to T0. It should be noted that healthy sows were used in this study, and the measured values therefore indicate that this reaction is physiological, which supports the results of Kaiser et al. (2018) [15]. These results suggest that, as an indicator of inflammatory processes around the time of birth, haptoglobin already increases significantly at an earlier stage.

Rectal temperature is used by veterinarians and farmers as a diagnostic tool to detect inflammatory processes in the body [1]. This was elevated at all time points after giving birth compared to before birth. It thus correlates with the concentrations of haptoglobin and pig-MAP, which also increased after giving birth and serve as indicators of inflammatory reactions in the body. However, it should be noted that lactational hyperthermia also influences rectal temperature and could contribute to the elevated temperatures [29]. Throughout the study, 12 rectal temperatures above 39.5 °C were recorded despite only clinically healthy sows being included. Therefore, APPs could be utilised as additional indicators of inflammatory events to prevent animals from being mistakenly diagnosed based solely on an elevated temperature.

Given that Swine Inflammation and Necrosis Syndrome (SINS) has been associated with systemic inflammatory and metabolic alterations [23], similar inflammatory pathways may be involved. APPs may therefore also have potential as biomarkers for the early detection of such conditions. However, dedicated studies are required to evaluate their diagnostic value and to establish disease-specific reference ranges.

### 4.5. Comparison of Serum, Saliva and Milk Concentrations at T0–T5 vs. T6

Undefined events may have an influence on APP changes even before birth; therefore, T6 was used as the basis for comparison. At T6, the sows were 14 days postpartum and therefore the influences of pregnancy and birth were already subsiding, even if this time point is not representative of a healthy, non-pregnant, non-lactating sow.

The serum concentrations of pig-MAP and haptoglobin were significantly increased at T4 and T5 compared to T6. In addition, serum haptoglobin levels were also higher at T3 compared to T6. Thus, the concentrations at these time points are also significantly increased compared to T0. Based on these results, the time points T4 (48 h p.p.) and T5 (72 h p.p.) are relevant for future investigations.

The pig-MAP and haptoglobin levels in milk were significantly increased at all time points (T1–T5) compared to T6. The results show a clear change in APP concentration in milk between birth and three days postpartum, followed by a decrease until day 14 postpartum. As mentioned above, the precise timing of the increase and decrease in APP concentrations in milk is not known. A decrease in milk concentrations is expected to occur between T5 (3 d p.p.) and T6 (14 d p.p.). Knowing the exact time is important for identifying sows with delayed decreases in APP concentrations due to concomitant diseases.

Haptoglobin in saliva showed a significant increase at T5 compared to T6, indicating that it rises markedly later than haptoglobin in serum and milk.

### 4.6. Comparison of Serum, Saliva and Milk Concentrations of APPs

The correlation coefficients between both APP concentrations in all three body fluids showed that the APP concentrations in milk, saliva and serum do not correlate over all of the time points. It is therefore important to establish individual reference values for different types of samples, rather than assuming that they will increase at the same rate.

### 4.7. Natural Variance in the Courses of the APPs

As shown in our study, even among clinically healthy sows, there can be significant variations in APP concentrations after birth. Sows 3, 15, 18 and 19 showed conspicuous differences in pig-MAP levels, with sows 3, 18 and 19 also showing elevated temperatures. Sow 4 also had elevated temperatures while sows 7, 8 and 14 showed slight increases but had no other symptoms. Consequently, further studies of sows with PPDS are needed to establish a cut-off point and determine the extent of APP concentration increases.

### 4.8. Limitations of the Study

Further studies are needed to determine the reference values for physiological increases in APP concentrations and to establish whether factors such as litter size, birth weight, birth duration and also induction of birth have a significant influence. This descriptive pilot study had a small sample size; therefore, further follow-up studies including both a control group and a group of clinically ill animals are necessary to clearly differentiate between healthy and diseased individuals. Without such differentiation, it cannot be excluded that subclinically infected animals may also have been included in our study.

## 5. Conclusions

This descriptive pilot study provides evidence regarding the timing and magnitude of the increase in APP levels in sows before and after parturition. However, further research is needed to determine the cut-off values for distinguishing between healthy and diseased sows. Premature increases or delayed decreases in concentrations could also indicate non-physiological processes.

This study is a cornerstone study as it is the first to measure pig-MAP levels in milk.

Notably, the results indicate that rectal temperature cannot be used as the sole indicator of PPDS, and that APPs could be a more suitable indicator. Future studies could explore whether collecting milk samples and performing ELISA analyses directly in the barn may improve the diagnosis of PPDS.

## Figures and Tables

**Table 1:** Parameters of clinical examination. Faecal consistency was measured using the scale of Oliviero et al. (2009) [24].

Parameter	Classification
General behaviour	Calm and alert, slightly reduced, moderately reduced, severely reduced
Breathing rate	[n/min]
Rectal temperature	[°C]
Appetite	Present, absent
Udder colour	Pale pink, slightly reddened, moderately reddened, highly reddened
Udder consistency	Soft and elastic, plump and elastic, plump
Udder temperature	[°C]
Faecal consistency	No faeces (0), dry and pellet-like (1), between dry and normal (2), normal and soft, but firm and well formed (3), between normal and moist, still formed but not firm (4), very moist faeces, unformed and liquid (5)

## Data Availability

The datasets used and analysed in the current study are available from the corresponding author on reasonable request.

## Appendix A

**Table A1:** Sow ages, number of litters, duration of birth and BCS.

Sow Number	Age	Number of Litters	Duration of Birth [Minutes]	BCS
2	1 year	1	300	3
3	1 year	1	180	3
4	1 year, 1 month	1	240	3.5
5	2 years, 2 months	3	310	4
6	3 years, 6 months	4	255	3.5
7	1 year, 9 months	2	120	3.5
8	1 year, 9 months	2	180	3
9	1 year, 9 months	2	255	4
10	1 year, 4 months	1	120	3.5
11	2 years, 5 months	4	205	3
12	2 years, 5 months	4	120	3.5
14	7 months	1	195	3
15	3 years, 5 months	7	420	3
16	2 years, 11 months	6	510	3.5
17	2 years, 2 months	4	-	3
18	2 years, 2 months	4	420	3
19	1 year, 8 months	3	185	3
20	1 year, 4 months	2	180	2
21	1 year, 8 months	3	420	3

**Table A2:** Means ± standard deviations and medians of haptoglobin (hp) and pig-MAP (pM) concentrations in serum (se), milk (m) [mg/mL] and saliva (sa) [µg/mL] at T0–6 and overall. Timepoints: T0 (112th day of pregnancy), T1 (intrapartum), T2 (12 h postpartum (p.p.)), T3 (24 h p.p.), T4 (48 h p.p.), T5 (72 h p.p.) and T6 (14 d p.p.).

APP			T0–T6	T0	T1	T2	T3	T4	T5	T6
HP	se	Mean ± sd	2.917 ± 1.292	2.027 ± 0.710	2.070 ± 0.725	2.888 ± 0.932	3.261 ± 1.099	3.933 ± 1.392	3.885 ± 1.488	2.340 ± 0.935
		Median	2.666	1.977	2.025	2.901	2.788	3.688	3.472	2.325
	m	Mean ± sd	0.649 ± 0.600	-	1.008 ± 0.577	0.690 ± 0.422	0.786 ± 0.545	0.735 ± 0.782	0.596 ± 0.595	0.092 ± 0.081
		Median	0.467	-	0.882	0.598	0.739	0.411	0.351	0.094
	sa	Mean ± sd	3.420 ± 7.143	2.073 ± 5.677	2.349 ± 2.272	4.051 ± 4.719	4.150 ± 8.926	3.240 ± 3.383	7.158 ± 13.746	0.908 ± 0.794
		Median	1.430	0.360	1.690	2.120	1.640	2.230	2.710	0.635
PM	se	Mean ± sd	2.194 ± 1.602	1.165 ± 0.478	1.318 ± 0.616	1.971 ± 1.321	2.422 ± 1.117	3.218 ± 1.835	3.585 ± 2.301	1.677 ± 1.087
		Median	1.871	1.116	1.175	1.794	2.126	2.554	2.535	1.397
	m	Mean ± sd	0.050 ± 0.028	-	0.061 ± 0.017	0.044 ± 0.008	0.040 ± 0.014	0.062 ± 0.028	0.071 ± 0.040	0.015 ± 0.010
		Median	0.047	-	0.060	0.044	0.041	0.051	0.058	0.010

**Table A3:** Serum, milk and saliva concentrations of haptoglobin and pig-MAP at time points T1–T6 compared to time point T0. Significance codes: <0.001 ‘***’ and <0.01 ‘**’; timepoints: T0 (112th day of pregnancy), T1 (intrapartum), T2 (12 h postpartum (p.p.)), T3 (24 h p.p.), T4 (48 h p.p.), T5 (72 h p.p.) and T6 (14 d p.p.).

Time Point	Serum HPT	Saliva HPT	Serum Pig-MAP	Rectal Temperature
T0–T1	0.993	1.000	0.982	0.386
T0–T2	<0.002 **	0.888	0.087	<0.001 ***
T0–T3	<0.001 ***	0.872	0.004 **	<0.001 ***
T0–T4	<0.001 ***	0.960	<0.001 ***	<0.001 ***
T0–T5	<0.001 ***	0.154	<0.001 ***	<0.001 ***
T0–T6	0.783	0.993	0.728	<0.001 ***

**Table A4:** Serum, milk and saliva concentrations of haptoglobin and pig-MAP at time points T1–T5 compared to time point T6. Significance codes: <0.001 ‘***’, <0.01 ‘**’ and <0.05 ‘*’; timepoints: T0 (112th day of pregnancy), T1 (intrapartum), T2 (12 h postpartum (p.p.)), T3 (24 h p.p.), T4 (48 h p.p.), T5 (72 h p.p.) and T6 (14 d p.p.).

Time Point	Serum HPT	Milk HPT	Saliva HPT	Serum PIG-MAP	Milk Pig-MAP
T6–T0	0.778	-	0.994	0.723	-
T6–T1	0.907	<0.001 ***	0.964	0.917	<0.001 ***
T6–T2	0.221	<0.001 ***	0.582	0.874	0.002 **
T6–T3	0.002 **	<0.001 ***	0.545	0.128	0.006 **
T6–T4	<0.001 ***	<0.001 ***	0.711	<0.001 ***	<0.001 ***
T6–T5	<0.001 ***	0.002 **	0.018 *	<0.001 ***	<0.001 ***

**Table A5:** *p*-values and cor-values of correlations between serum, milk and saliva concentrations of pig-MAP and haptoglobin. Significance code *p*-values: <0.001 ‘***’ and <0.05 ‘*’; T0 (112th day of pregnancy), T1 (intrapartum), T2 (12 h postpartum (p.p.)), T3 (24 h p.p.), T4 (48 h p.p.), T5 (72 h p.p.) and T6 (14 d p.p.).

Correlation Cor-Value	T0–T6	T0	T1	T2	T3	T4	T5	T6
Pig-MAP Serum & Milk	0.712	-	0.281	−0.404	0.048	0.688	0.963	0.829
HPT Serum & Milk	0.209	-	0.243	0.081	0.074	0.527	0.181	−0.114
HPT Serum & Saliva	0.102	−0.080	0.236	−0.209	−0.156	0.002	0.069	0.218
HPT Milk & Saliva	0.128	-	0.115	0.211	0.165	−0.270	0.187	0.333
**Correlation *p*-Value**	**T0–T6**	**T0**	**T1**	**T2**	**T3**	**T4**	**T5**	**T6**
Pig-MAP Serum & Milk	<0.001 ***	-	0.500	0.320	0.911	0.059	<0.001 ***	0.021 *
HPT Serum & Milk	0.031 *	-	0.347	0.751	0.763	0.025 *	0.487	0.652
HPT Serum & Saliva	0.278	0.759	0.398	0.420	0.551	0.996	0.792	0.386
HPT Milk & Saliva	0.210	-	0.683	0.416	0.526	0.350	0.489	0.177
